# Hypoxia Correlates With Poor Survival and M2 Macrophage Infiltration in Colorectal Cancer

**DOI:** 10.3389/fonc.2020.566430

**Published:** 2020-11-20

**Authors:** Lina Qi, Jiani Chen, Yanmei Yang, Wangxiong Hu

**Affiliations:** ^1^ Cancer Institute (Key Laboratory of Cancer Prevention and Intervention, China National Ministry of Education), The Second Affiliated Hospital, Zhejiang University School of Medicine, Hangzhou, China; ^2^ Key Laboratory of Reproductive and Genetics, Ministry of Education, Women’s Hospital, Zhejiang University School of Medicine, Hangzhou, China

**Keywords:** colorectal cancer, HIF-1, hypoxia, *KRAS* mutation, M2 macrophages

## Abstract

**Background:**

It is widely accepted that the oxygen level in tumor tissue is significantly lower than the adjacent normal tissue, thus termed hypoxia. Intratumoral hypoxia represents a major driving force in cancer progression, recurrence, metastasis, and decreased survival. Though multiple gene signatures reflect the complex cellular response to hypoxia have been established in several cancer types such as head and neck, breast, and lung cancers, the hypoxic panorama in colorectal cancer (CRC) remains poorly understood.

**Methods:**

A hypoxic signature constituted by a total of 356 genes, including canonical hypoxia-responsive ADM, ANGPTL4, CA9, and VEGFA, was established based on systemic literature search. A total of 1,730 CRC samples across four independent cohorts were used for *nonnegative matrix factorization clustering and subtyping.* Prognosis, molecular signatures, pathways, and tumor-infiltrating lymphocytes were compared between the subtypes.

**Results:**

CRCs mainly fell into two subgroups, one indicated as hypoxia and the other one designated as normoxia. Hypoxia was correlated with poor outcomes in CRC and will increase the risk of a subset of stage II patients to the level of normoxic stage III. Additionally, hypoxia was closely associated with activation of RAS signaling pathway independent of *KRAS* mutation. More M2 macrophage infiltration was another hypoxic marker indicated that subsets of patients with high M2 macrophages may benefit from macrophage-targeting therapy.

**Conclusions:**

These findings will facilitate the development of a hypoxia-oriented therapy strategy to enhance the treatment effect in the near future.

## Introduction

Tumor hypoxia is correlated with advanced progression, treatment resistance and poor clinical outcomes (1, 2). It is widely accepted that the oxygen level in hypoxic tumor tissue is significantly lower than the oxygenation of the respective normal tissues and on average it is between 1%–2% O_2_ and below (3). Intratumoral hypoxia is a well established resistance factor for radiotherapy and is increasingly recognized as promoting resistance to systemic cancer therapies. Hypoxia promotes a more aggressive and resistant cancer phenotype, primarily mediated by hypoxia-inducible factor 1 (HIF-1), a transcription factor that is stable only in low-oxygen condition, which leads to cell cycle arrest, angiogenesis, and accelerated glycolysis (3). Nevertheless, tumor oxygen level largely depends on the initial oxygenation of the tissue, the size and stage of the tumor, and the method of oxygen measurement. In addition, different measurement methods often yield discrepant result and diagnose methods such as oxygen electrode and phosphorescence quenching and near-infrared spectroscopy are tedious and not suit to large numbers of samples.

Given the close relationship between hypoxia and cancer progression and metastasis, multiplex markers such as gene signatures potentially better reflect the complex cellular response to hypoxia have been established in several solid tumor types such as head and neck, breast, and lung cancers (4, 5). However, the hypoxia-inducible gene signature in colorectal cancer (CRC) remains poorly understood.

Here, we use TCGA, GSE14333, GSE17538, and GSE39582 four independent cohorts to explore the potential clustering of CRC samples based on manually curated hypoxia markers. We found that hypoxia correlated with poor outcomes in CRC. In addition, hypoxia is closely associated with activation of RAS signaling pathway independent of *KRAS* mutation and M2 macrophage polarization.

## Materials and Methods

### Multi-Omic Data for CRCs in TCGA

CRC somatic mutational profiles and clinical information were downloaded from The Cancer Genome Atlas (TCGA) data portal (06/02/2018). Silent mutations, RNA mutations, and any mutation located within the intron, flanking sequence, 5’ untranslated region (UTR), and 3’UTR were discarded. Then, clinical information of each patient was added to mutational information *via* unique sample ID. *This study was approved by the Ethics Committee of the Second Affiliated Hospital, School of Medicine, Zhejiang University.*

### Curation of Hypoxic Signature

To build a robust hypoxia signature, we collected a list of well-annotated gene expression signatures across different cancer types, including the well-known pancancer hypoxia 15-gene (5), 26-gene hypoxia signature in laryngeal cancer (6), 24-gene hypoxia signature in high-risk bladder cancer (7), 20-gene that showed the greatest fold induction following hypoxic exposure in MCF7 cells (8), 28-gene hypoxia-related prognostic signature for localized prostate cancer (9), hypoxia gene expression classifier in head and neck cancer (4, 10), 27-gene that was found as hypoxia induced, pH unaffected in human squamous cell carcinomas (11), nine-gene derived from Caco-2 CRC cells in response to hypoxia (12), and 200-gene under hypoxia hallmark in Molecular Signatures Database (MSigDB) (13). Then, a hypoxia-signature constituted by a total of 356 genes (**Table S1**), including canonical hypoxia-responsive ADM, ANGPTL4, CA9, and VEGFA, was used for subsequent non-negative matrix factorization (NMF) clustering.

### Gene Expression Data Processing and Normalization

All level 3 tumor RNASeqV2 mRNA expression datasets were obtained from TCGA (October 2015). Genes with expression levels < 1 (RSEM-normalized counts) in more than 50% samples were removed. The GSE14333, GSE17538, and GSE39582 (Affymetrix HG U133 Plus 2.0 arrays) datasets were downloaded from the Gene Expression Omnibus (GEO, https://www.ncbi.nlm.nih.gov/geo/). Raw CEL files were processed using the *affy* package of BioConductor (14). Then, MAS5 algorithm was used for background correction, normalization, and summarization of single probes for all probe sets, which was performed similar as in our previous work (15). NMF was performed using the *NMF* package for R (16). Differentially expressed genes (DEGs) were identified using the *DEGSeq* package for R/Bioconductor according to a false discovery rate (FDR)-adjusted *P* value < 0.05 and fold change > 2 conditions (17). Gene ontology (GO) and Kyoto Encyclopedia of Genes and Genomes (KEGG) enrichment analyses were performed using the *clusterProfiler* package from BioConductor (18). Significantly enriched GO terms and pathways were selected according to an FDR-adjusted *P* value < 0.05.

### Functional Enrichment in CRC Under Hypoxia

Hallmark gene sets from molecular signatures database were used for determining whether any signatures were enriched under hypoxic condition by gene set enrichment analyses (GSEA) (19). Significantly enriched hallmarks were selected according to a FDR q-value < 0.05.

### Survival Analysis

Survival differences between the hypoxic and normoxic groups were tested by the Kaplan-Meier method and analyzed with the log-rank test with functions *survfit* and *survdiff* in the *survival* package for R (20). Cox univariate model was performed with function *coxph* in the R package *survival*. A *P* value < 0.05 was considered significant.

### Deciphering Tumor Infiltrated Lymphocytes (TILs) in CRCs

To quantify the relative amount of distinct TILs, CIBERSORT was used to calculate the proportions of 22 lymphocytes in tumor tissue (21). The permutations were set to >=100, and quantile normalization (QN) of the input expression mixture was set to FALSE for TCGA RNAseq data. Samples with CIBERSORT *P* value > 0.05 were discarded from further comparison.

## Results

### Classification of CRCs Based on Hypoxic Gene Expression Signature

**Figure 1:**
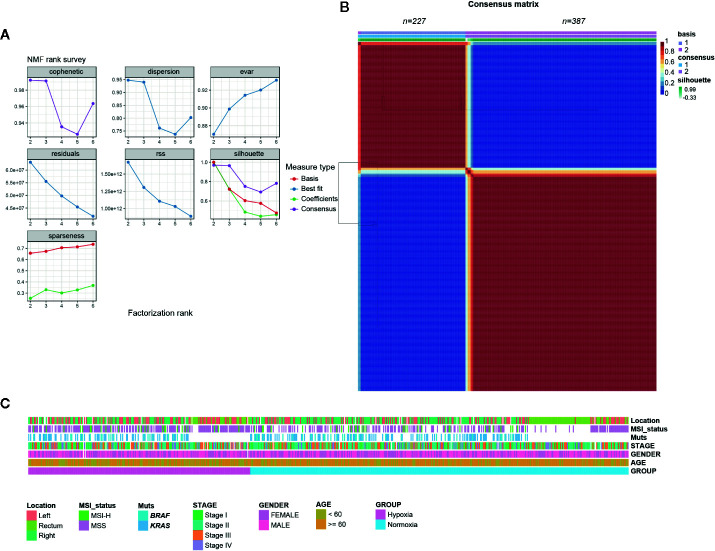
Colorectal cancer (CRCs) clustered into two gene expression-based subtypes based on hypoxic signature. **(A)** Rank survey of the parameter *r* in non-negative matrix factorization (NMF). **(B)** Clustering of 617 CRCs in the The Cancer Genome Atlas (TCGA) by NMF. **(C)** Association of clinical characteristics and hypoxic subtypes.

NMF consensus clustering of 614 TCGA CRC samples using the established 356 gene hypoxia signature revealed that it mainly fell into two subgroups, one indicated as hypoxia hereafter and the other one designated as normoxia (**Figures 1A, B**). To validate the two subgroups identified in TCGA cohort, we also examined three other datasets GSE39582 (French Ligue Nationale Contre le Cancer), GSE17538 (61 from Vanderbilt Medical Center and 177 patients from the Moffitt Cancer Center), and GSE14333 (Royal Melbourne Hospital, Western Hospital and Peter MacCallum Cancer Center in Australia, and the H. Lee Moffitt Cancer Center in the United States), each with more than 200 CRC samples. Consistent with the findings in TCGA, the two hypoxia-driven subgroups held true in all of the three GEO datasets, suggesting the discriminative robustness of our collected hypoxic signature.

Considering some well-established factors such as *KRAS*/*BRAF* mutation, microsatellite stable (MSS)/microsatellite instability (MSI) status, right-sided colon cancer (RCC), and American Joint Committee on Cancer (AJCC) stages are associated with CRC patients’ outcomes, we tried to determine whether hypoxia tend to correlate with above indicators. Somewhat unexpectedly, no biased tendency of *KRAS*/*BRAF* mutation, MSS/MSI, and stage distributions under hypoxia was observed (**Figure 1C**). However, we found that younger patients (< 60 year) had a preference with hypoxia, while an opposite tendency was observed for RCC in TCGA samples (**Figure 1C**, **Table S2**).

### Hypoxia Was Correlated With Poor Outcomes

**Figure 2:**
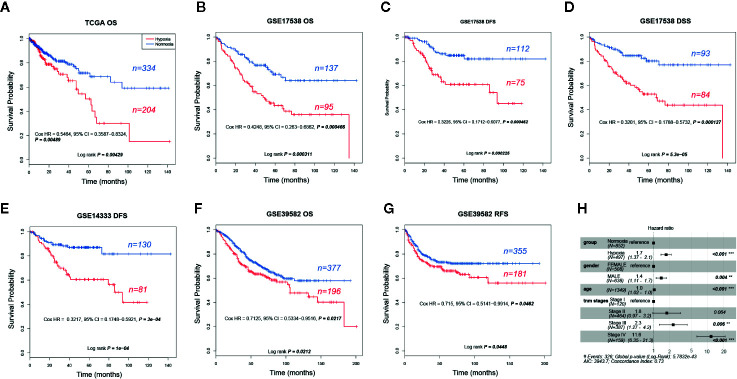
Prognostic analysis of hypoxic and normoxic groups in The Cancer Genome Atlas (TCGA), GSE14333, GSE17538, and GSE39582 datasets. **(A)** KM plot of overall survival (OS) status for TCGA samples. **(B)** KM plot of OS status for GSE17538 samples. **(C)** KM plot of disease free survival (DFS) status for GSE17538 samples. **(D)** KM plot of disease-specific survival (DSS) status for GSE17538 samples. **(E)** KM plot of DFS status for GSE14333 samples. **(F)** KM plot of OS status for GSE39582 samples. **(G)** KM plot of relapse-free survival (RFS) status for GSE39582 samples. **(H)** OS hazard ratio of different clinical characteristics based on pooled TCGA, GSE17538, and GSE39582.

Given that hypoxia tends to associate with aggressive phenotypes, we then asked whether clinical outcomes differed greatly between these two groups. As our expected, hypoxia led to an obvious shorter OS and earlier relapse or progression in CRC. We found that hypoxia was significantly correlated with unfavorable overall survival (OS) in TCGA (five-year survival, 0.5 vs. 0.72, 95% confidence interval (CI) 0.37–0.69 vs. 0.63–0.81), GSE17538 (0.43 vs. 0.7, 95% CI 0.33–0.55 vs. 0.61–0.79), and GSE39582 (0.63 vs. 0.71, 95% CI 0.57–0.71 vs. 0.67-0.77) and adverse disease-specific survival (DSS) in GSE17538 (0.53 vs. 0.8, 95% CI 0.42–0.66 vs. 0.71–0.9). Furthermore, hypoxia led to poorer disease free survival (DFS) or relapse-free survival (RFS) was observed in GSE17538 (0.61 vs. 0.85, 95% CI 0.5-0.75 vs. 0.78-0.93), GSE14333 (0.61 vs. 0.87, 95% CI 0.5–0.74 vs. 0.81–0.93), and GSE39582 (0.66 vs. 0.73, 95% CI 0.59–0.74 vs. 0.69–0.78, **Figures 2A–G**). In addition, hypoxia was an independent significant OS prognostic factor in CRC with a hazard ratio of 1.7 (95% CI 1.37–2.1) compared to the normoxic group (**Figure 2H**). In view of the score of hazard ratio, hypoxia contributed more to the prognosis than gender and TNM stage II (**Figure 2H**).

### Increased Risk of a Subset of Stage II CRCs Under Hypoxic Condition

**Figure 3:**
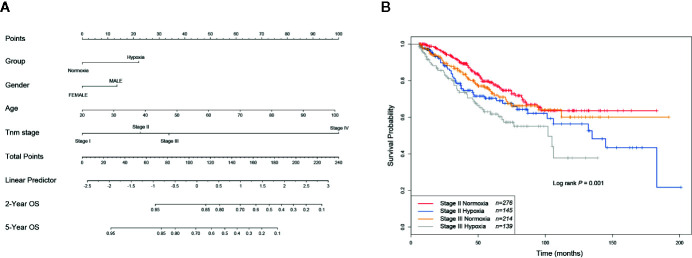
Hypoxia is linked to increased risks of a subset of stage II colorectal cancer (CRCs). **(A)** Nomogram showed that hypoxia had a higher weigh than the difference between stage II and III. **(B)** KM plot showed that hypoxic stage II CRCs even had a worse prognosis than the normoxic stage III group.

As hypoxia potentially had a larger effect to poor survival of CRCs (**Figure 2H**), to better quantify the weight of known survival factors, a nomogram model was constructed to solve this problem. Nomogram showed that hypoxia had a higher risk than the score between stage II and stage III (**Figure 3A**), we thus attempted to understand whether a part of high-risk stage II CRCs were hypoxia-related. Then, stage II and III CRC patients were stratified into the hypoxic and normoxic groups. Kaplan-Meier plot showed that the hypoxic group had a significant poorer survival than the normoxic group in both stage II and III, hypoxic stage II CRCs even had a worse prognosis than the normoxic stage III group (five-year survival, stage II normoxia 0.79, 95% CI 0.74–0.85; stage II hypoxia 0.70, 95% CI 0.62–0.79; stage III normoxia 0.73, 95% CI 0.66–0.81; stage III hypoxia 0.62, 95% CI 0.53–0.72; Log rank *P* = 0.001, **Figure 3B**). This result indicated that hypoxia was a high-risk factor for a subset of stage II CRC patients, like big tumor size, vascular, and lymphatic vessel invasion, and may be a clinical index that should be considered for adjuvant therapy in future clinical practice.

### Hypoxia-Associated Molecular Signatures in CRC

**Figure 4:**
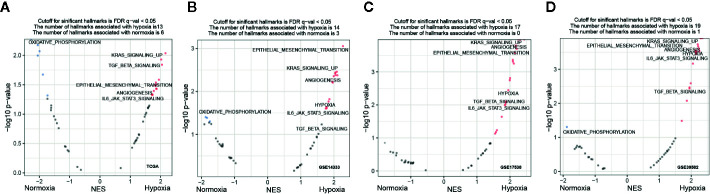
Functional enrichment signatures of colorectal cancer (CRCs) under hypoxic conditions. **(A–D)** Gene set enrichment analyses (GSEA) enrichment hallmarks visualized as volcano plots in The Cancer Genome Atlas (TCGA), GSE14333, GSE17538, and GSE39582, respectively. Hallmarks significantly correlated with hypoxia or normoxia were labeled with red and blue dots, respectively. NES, normalized enrichment score.

To explore whether specific hallmark signatures were enriched subject to hypoxia that associated with poor outcomes, GSEA was performed to determine the hypoxia-oriented molecular characteristics. Intriguingly, we found majority of significant signatures were concerned with hypoxia (**Figure 4A**). Aggressive tumor features, such as KRAS signaling up (NES = 2.04, FDR q-value = 0.014), EMT (NES = 1.94, FDR q-value = 0.026), myogenesis (NES = 1.82, FDR q-value = 0.033), apical junction (NES = 1.87, FDR q-value = 0.037), angiogenesis (NES = 1.73, FDR q-value = 0.047), IL6/JAK/STAT3 pathway (NES = 1.71, FDR q-value = 0.047), and TGFβ signaling (NES = 2.02, FDR q-value = 0.012) were the most significantly enriched hallmarks under hypoxia (**Figure 4A**). Contrast to the hypoxic subgroup, oxidative phosphorylation (NES = -2.27, FDR q-value = 0) was the most significant hallmark in the normoxic subgroup. These trends were basically held in all other three datasets GSE14333 (**Figure 4B**), GSE17538 (**Figure 4C**), and GSE39582 (**Figure 4D**).

To better clarify the underlying mechanism of tumor adaption to hypoxia, we sought to identify the DEGs between the two subtypes. Intriguingly, majority of DEGs were upregulated by hypoxia in all examined datasets: GSE14333 (85.5%, 506 out of 592 DEGs were upregulated by hypoxia), GSE17538 (95.6%, 326 out of 341 DEGs were upregulated by hypoxia), GSE39582 (90.6%, 778 out of 859 DEGs were upregulated by hypoxia), and TCGA (76.7%, 1,013 out of 1,320 DEGs were upregulated by hypoxia). GO enrichment interrogation based on the hypoxia-upregulated DEGs revealed that hypoxia was closely associated with extracellular matrix/structure organization and collagen fibril organization (**Figure S1**). KEGG pathway enrichment further confirmed the significant association of hypoxia with ECM-receptor interaction, focal adhesion, and cell adhesion molecules (**Figure S1**).

### Activation of RAS Signaling Under Hypoxia Was Independent of *KRAS* Mutation

**Figure 5:**
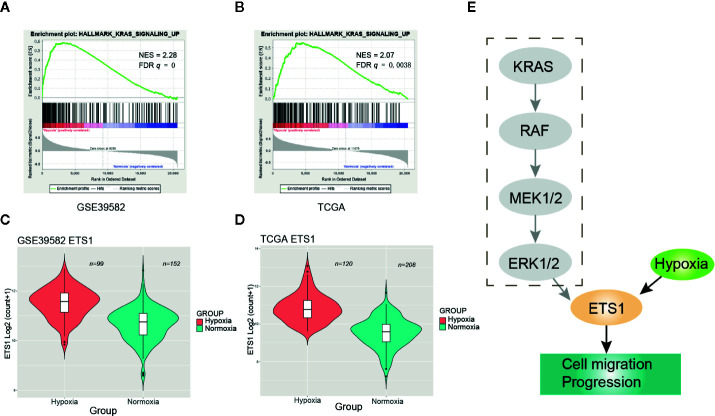
Hypoxia may activate the RAS signaling pathway through up-regulating ETS1. **(A)** Enrichment plot of RAS signaling up in GSE39582 under hypoxic condition. **(B)** Enrichment plot of RAS signaling up in The Cancer Genome Atlas (TCGA) under hypoxic condition. **(C)** ETS1 had a significant higher expression level in the hypoxic group than the normoxic group using GSE39582 dataset. **(D)** ETS1 had a significant higher expression level in the hypoxic group than the normoxic group using TCGA dataset. **(E)** Schematic diagram of KRAS pathway activation by hypoxia-induced upregulation of EST1 in *KRAS* WT CRCs.

GSEA revealed that activation of RAS signaling under hypoxia was one of the most significant signatures (**Figure 4**). However, no significant frequency of *KRAS* mutational difference was observed between the hypoxic and normoxic groups (**Figure 1C**). Then, we speculated that hypoxia activated the RAS signaling casade without *KRAS* mutation. To test our hypothesis, we performed clustering of CRC samples with wildtype (WT) *KRAS* status. Intriguingly, we found that CRCs with WT *KRAS* also fell into two groups, the proportion of hypoxia and normoxia was consistent with the abovementioned whole CRC cohort. GSEA consolidated the activation of RAS signaling in the hypoxic group without *KRAS* mutation both in GSE39582 (NES = 2.28, FDR *q* = 0, **Figure 5A**) and TCGA (NES = 2.07, FDR *q* = 0.0038, **Figure 5B**) cohorts. Additionally, ETS1, a genome-wide effector of RAS/ERK signaling in epithelial cells and involvement in the upregulation of hypoxia-inducible genes (22, 23), was found significantly unregulated in the hypoxic group compared to the normoxic group (**Figures 5C, D**, **Table S3**). These results indicated that RAS signaling pathway may be triggered by ETS1 in a *KRAS* mutation-independent manner by hypoxia (**Figure 5E**), suggesting that CRC patients with WT *KRAS* status should consider hypoxia-target therapy or combination with EGFR inhibitor if failure of mono-EGFR inhibitor treatment.

### Hypoxia Was Associated With More M2 Macrophage Infiltration

**Figure 6:**
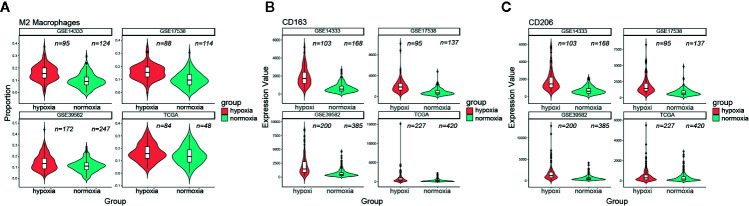
Hypoxia correlated with more M2 macrophage infiltration. **(A)** Comparison of M2 macrophage infiltration proportion between the hypoxic and normoxic groups. **(B)** CD163 had a significant higher expression level in the hypoxic group than in the normoxic group. **(C)** CD206 had a significant higher expression level in the hypoxic group than in the normoxic group.

Considering the unfavorable outcomes and aggressive phenotype under hypoxic conditions, we then asked whether the tumor immune microenvironment (TIME) differed greatly between these two groups. CIBERSORT was used to quantify the relative amount of 22 tumor lymphocytes in all four CRC datasets. Of note, CD8 T cell and CD4 memory resting T cell were less infiltrated under hypoxic conditions (**Figure S2**). On the contrary, we found that hypoxia was significantly associated with neutrophil and macrophage infiltration and polarization. Both M0 macrophages and M2 macrophages were much more infiltrated under hypoxic conditions (e.g., M2 macrophages median 15.5% versus 11.5%, GSE14333; 15.4% versus 9.6%, GSE17538; 13.2% versus 11%, GSE39582; 15.7% versus 13%, TCGA; **Figure 6A**), however, this trend was not held for M1 macrophages. In addition, the aggregate amount of M2 macrophages was much larger than M1 macrophages (**Figure S2**). Two canonical markers of M2 macrophages, CD163 and CD206, had much higher expression levels in the hypoxic subgroup than in the normoxic subgroup in all four datasets (**Figures 6B, C**). Further correlation exploration between different lymphocytes observed that M2 macrophages had a negative correlation with plasma cells (**Figure S3**). TAMs were reported to promote EMT of CRC cells *via* IL6/JAK/STAT3 pathway (24). Given IL6/JAK/STAT3 was featured under hypoxic condition (**Figure 4**), it is tempting to believe that it had a close association with more M2 macrophage infiltration. IL6ST (gp130), the receptor for IL6 initiating signal transmission, exhibited a significant positive correlation with M2 macrophage infiltration under hypoxic conditions (**Figure S4**). Thus, the suppressive TIME orchestrated by hypoxia further exacerbated the tumor malignancy and, undoubtedly, a worse outcome.

## Discussion

Hypoxia is characteristic for solid tumors and has been known to contribute to tumor aggressive phenotypes including enhanced motility, invasiveness, and immune escape (25). In this study, we systematically analyzed four independent datasets covering different races and found that hypoxia was widely present in CRC. Hypoxic CRCs was characterized by enhanced angiogenesis, EMT, TGF-beta signaling, and undoubtedly, had worse OS, DFS, DSS, and RFS compared with the normoxic group. Notably, activation of the RAS signaling was one of typical characteristics under hypoxia and this association may through EST1 to activate the RAS pathway in a non-KRAS mutation-dependent manner to promote tumor proliferation and metastasis. In other words, not all CRCs with WT *KRAS* were suitable for solely cetuximab treatment. The mechanism of cetuximab resistance in *KRAS*-WT CRC patients remains poorly understood (26). It’s reported that increased DDX3 promoted by HIF-1α could induce cetuximab resistance *via* YAP1/SIX2 axis in *KRAS*-WT CRC cells (27). These evidences coupled with our findings indicated that in combination of hypoxia-targeted inhibitors such as TH-302 (a hypoxia-activated prodrug) may enhance drug susceptibility in cetuximab resistant *KRAS*-WT CRC. In this case, patients with WT *KRAS* may still need to determine oxygenation status before applying EGFR inhibitors since activation of the RAS signaling pathway *via* hypoxia may lead to EGFR inhibitor treatment failure according to our finding.

Furthermore, another interesting finding in this study was the hypoxic group of stage II CRCs had a worse outcome than the normoxic group of stage -II and even –III (**Figure 3B**). This finding provided a novel stratified basis for future CRC clinical treatment. In current clinical practice, postoperative adjuvant chemotherapy is required when stage II CRC patients have high-risk factors such as pT4 Tumors, obstruction or perforation, and lymphovascular and perineural invasion (28). Hypoxia, in this context, may also be taken into consideration when applying preoperative or postoperative adjuvant chemotherapy for selected stage II CRC patients.

It is also important to keep in mind that a better understanding of the interactions within the hypoxic tumor microenvironment is crucial for developing optimal new combination strategies. Through interrogating the constitution of TILs within CRCs, more M2 macrophage infiltration and a significant correlation with IL6/JAK/STAT3 pathway were revealed in the hypoxic group (**Figures 4** and **6**). Macrophages are the main infiltrating immunosuppressive cells within the tumor microenvironment (29). The M2 macrophage has been reported to promote tumor proliferation, angiogenesis (30), metastasis (31), and resistance to anti-cancer therapies (32). In breast cancer and pancreatic adenocarcinoma, hypoxia has been shown to positively regulate the expression of CD47, leading to cancer cell escape from phagocytosis mediated by macrophages (33). Blockade of the well-known CD47-SIRPα “don’t eat me signal” using monoclonal antibodies increases macrophage-mediated phagocytosis and elimination of various solid tumors (34–36), however, this therapeutic schedule may be not suit to hypoxic CRC because the expression of CD47 showed uncorrelated trend with hypoxia. In non-small cell lung cancer (NSCLC), direct depletion of tumor-associated macrophages (TAM) by clodronate was sufficient to abrogate aerobic glycolysis and tumor hypoxia, thereby improving tumor response to anti-cancer therapies (37), this therapeutic regimen may be also fit for CRC but warrants further investigation. Hypoxic CRC patients may also benefit from sarilumab (IL-6 receptor (IL-6R)-blocking antibody), which is approved for treatment of Castleman syndrome by the FDA (38), since IL6/JAK/STAT3 pathway was closely associated with M2 macrophages although their implication in oncogenesis was less well characterized (24). Anyway, a safe, subtle, and flexible combination treatment should be designed in order to extend the clinical benefit of cancer therapy to high intratumoral hypoxic CRC patients.

The limitation of our study is the retrospective design of our analysis of gene expression data from public databases. However, the strength of this study is that different platforms (TCGA: RNAseq, GEO: microarray) and different cohorts (TCGA: USA, GSE14333: Australia, GSE17538: USA, GSE39582: France) yielded consistent results, which is likely to overcome underlying biases. Our study requires further validation in larger CRC patients by using protein expression data.

## Conclusions

Collectively, we revealed that hypoxia contributed to an unfavorable prognosis of CRC by activating RAS signaling pathway in a *KRAS* mutation independent manner and activating IL6/JAK/STAT3 signaling pathway *via* more M2 macrophage infiltration. These results suggested that before EGFR-targeted inhibitor was intentionally applied, it’s best to test oxygenation status in advance, especially for the young CRC patients (< 60y).

## Data Availability Statement

The datasets presented in this study can be found in online repositories. The names of the repository/repositories and accession number(s) can be found in the article/**Supplementary Material**.

## Author Contributions

Conception and design: WH and YY. Provision of study materials or patients: WH and YY. Collection and assembly of data: LQ and JC. Data analysis and interpretation: WH, LQ, and JC. Manuscript writing: All authors. All authors contributed to the article and approved the submitted version.

## Funding

This work was supported by the National Natural Science Foundation of China (grant number 81802883) and the Fundamental Research Funds for the Central Universities (grant number 2018FZA7012) to WH.

## Conflict of Interest

The authors declare that the research was conducted in the absence of any commercial or financial relationships that could be construed as a potential conflict of interest.
