# Supplementary material for: Hypoxia Correlates With Poor Survival and M2 Macrophage Infiltration in Colorectal Cancer
Source: Front Oncol. 2020 Nov 20;10:566430. doi: 10.3389/fonc.2020.566430 (PMC7714992; doi:10.3389/fonc.2020.566430)
Supplement: Supplementary file 1 [file DataSheet_1.pdf]

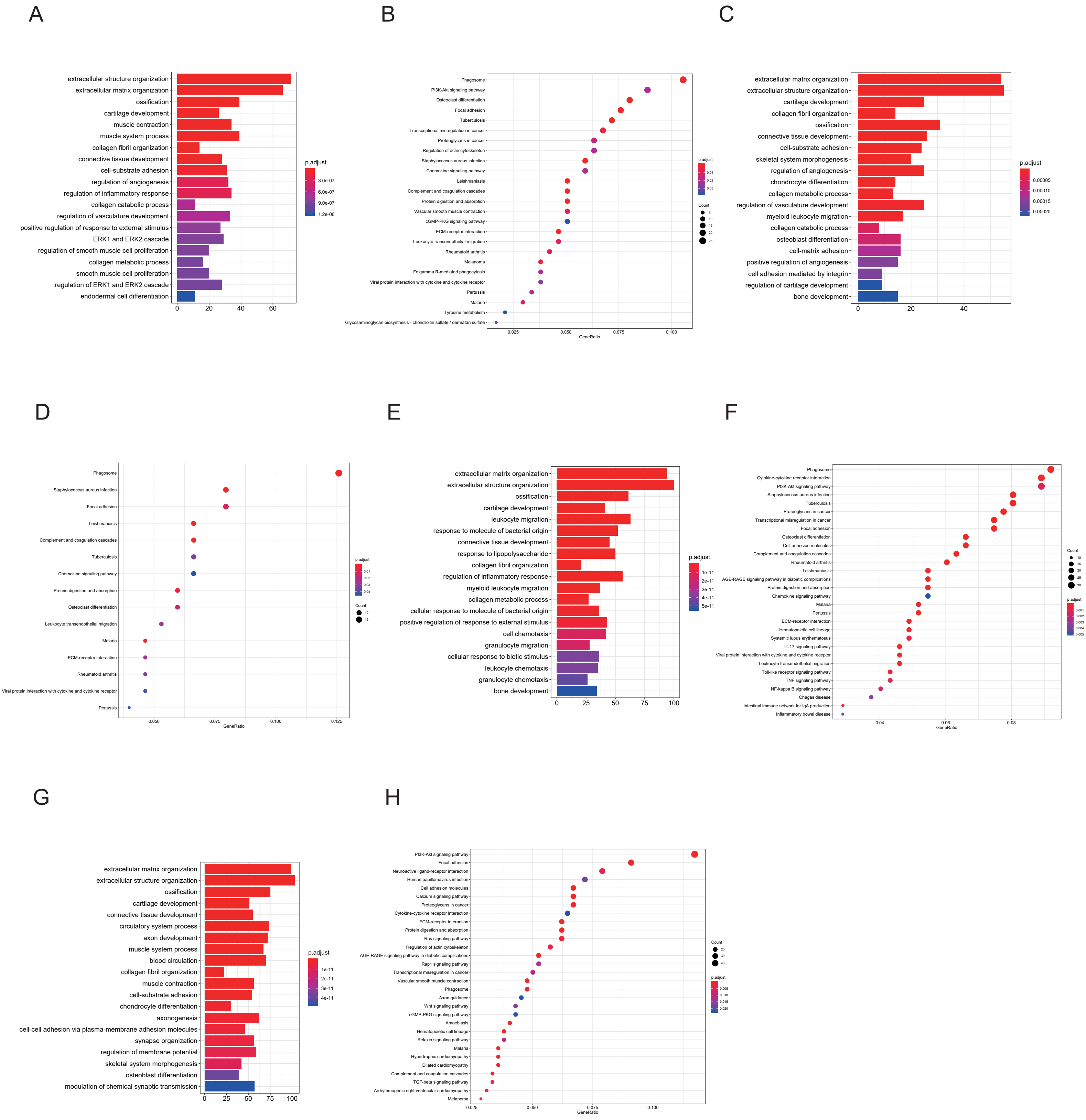

Figure S1. Enrichment of GO terms and KEGG pathways in GSE14333 (A and B), GSE17538 (C and D), GSE39582 (E and F), and TCGA (G and H) under hypoxic conditions.
