# Supplementary material for: Hypoxia Correlates With Poor Survival and M2 Macrophage Infiltration in Colorectal Cancer
Source: Front Oncol. 2020 Nov 20;10:566430. doi: 10.3389/fonc.2020.566430 (PMC7714992; doi:10.3389/fonc.2020.566430)
Supplement: Supplementary file 4 [file DataSheet_4.pdf]

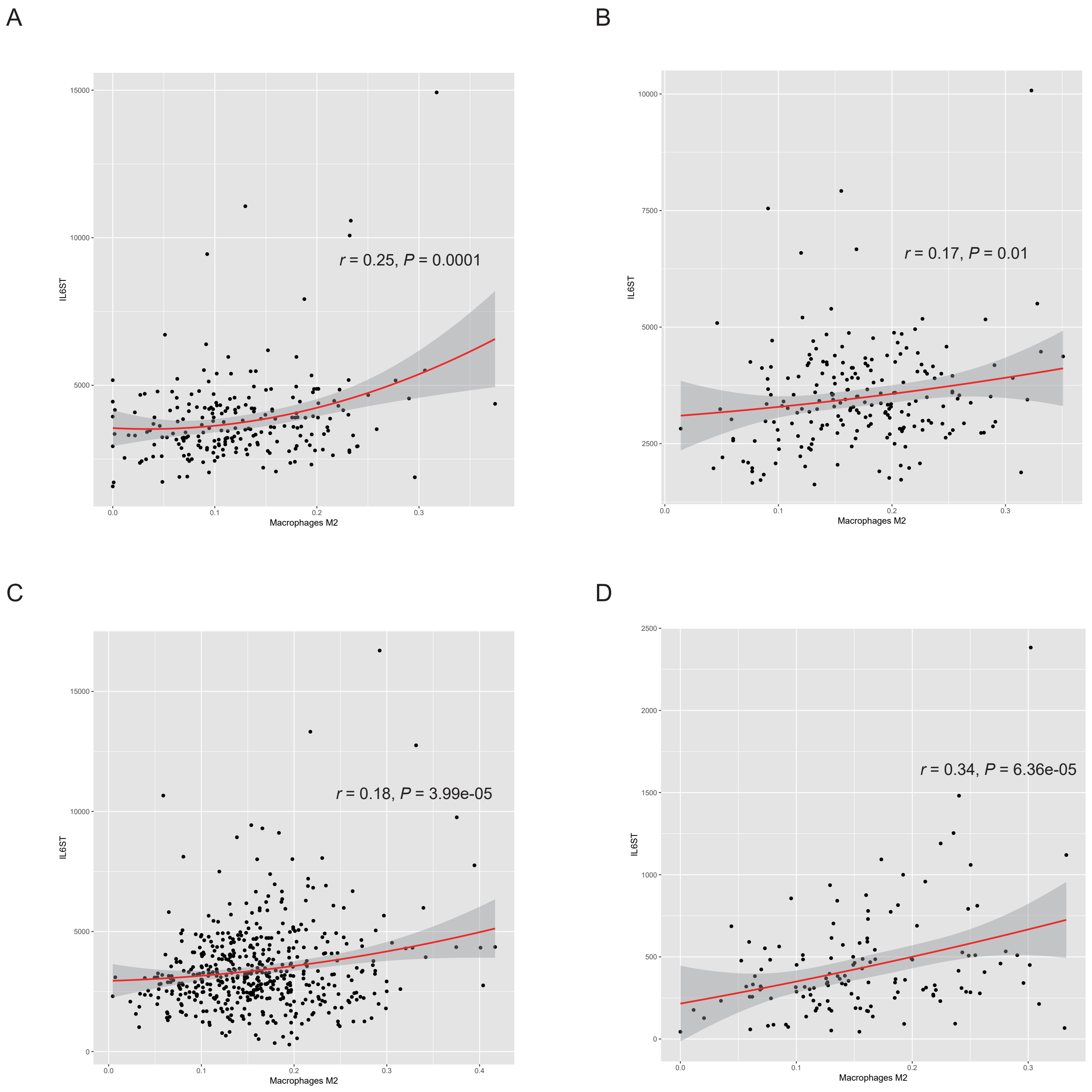

Figure S4. The correlation of IL6ST and M2 macrophage infiltration in GSE14333 (A), GSE17538 (B), GSE39582 (C), and TCGA (D), respectively.
