# Supplementary material for: Hypoxia Correlates With Poor Survival and M2 Macrophage Infiltration in Colorectal Cancer
Source: Front Oncol. 2020 Nov 20;10:566430. doi: 10.3389/fonc.2020.566430 (PMC7714992; doi:10.3389/fonc.2020.566430)
Supplement: Supplementary file 6 [file Table_2.docx]

| **Table S2. Clinical characteristics of hypoxic and normoxic CRC samples in TCGA.** | | | |
| --- | --- | --- | --- |
|  | **TCGA** | | |
|  | **Hypoxia (N=227)** | **Normoxia (N=387)** | ***P* value** |
| **Age** |  |  | **0.001** |
| <60 | 90 | 103 |  |
| ≥60 | 137 | 284 |  |
| **Gender** |  |  | 1 |
| Male | 118 | 206 |  |
| Female | 105 | 181 |  |
| NA | 4 | 0 |  |
| **TNM stage** |  |  | 0.2 |
| I | 33 | 71 |  |
| II | 145 | 257 |  |
| III | 0 | 0 |  |
| IV | 37 | 49 |  |
| NA | 12 | 10 |  |
| **Location** |  |  | **0.01** |
| Right | 83 | 170 |  |
| Left | 101 | 126 |  |
| NA | 43 | 91 |  |
| **KRAS** |  |  | 1 |
| Wild type | 22 | 37 |  |
| Mutated | 56 | 98 |  |
| NA | 149 | 252 |  |
| **BRAF** |  |  | 1 |
| Wild type | 56 | 98 |  |
| Mutated | 22 | 37 |  |
| NA | 149 | 252 |  |
| **Microsatellite status** |  |  | 0.500 |
| MSS | 156 | 163 |  |
| MSI-H | 25 | 33 |  |
| NA | 46 | 191 |  |
